# Anhedonia and Pessimism in Hospitalized Depressed Adolescents

**DOI:** 10.1155/2011/795173

**Published:** 2010-10-12

**Authors:** Zinoviy Gutkovich, Richard F. Morrissey, Ricardo K. Espaillat, Robert Dicker

**Affiliations:** ^1^Division of Child and Adolescent Psychiatry, Long Island Jewish Medical Center, Schneider Children's Hospital, NY 11040, USA; ^2^Center for Psychological Services, St. John's University, Seton Complex, 152-11 Union Turnpike, NY 11367, USA; ^3^Rega Mental Health Center, 5511 Sw 8th Street, Miami, FL 33134, USA; ^4^Long Island Jewish Medical Center, 269-01 76th Avenue, New Hyde Park, NY 11042, USA

## Abstract

This longitudinal study investigates whether anhedonia and pessimistic attributional style represent a clinical state or a trait in hospitalized depressed adolescents. 81 consecutive adolescent inpatients were screened with the Beck Depression Inventory (BDI) and the clinician-rated Major Depressive Disorder (MDD) criteria sheet. 51 patients with BDI score ≥10 and/or ≥4 symptoms on MDD criteria sheet were assessed at Time 1 upon admission, with 39 patients (78%) assessed at discharge (Time 2) with the Pleasure Scale for Children and Children's Attributional Style Questionnaire—Revised. Anhedonia and pessimism at admission were associated with BDI scores at admission and discharge as well as number of depressive symptoms and depression severity. MDD diagnosis was associated with anhedonia, but not with pessimism. Pessimism—but not anhedonia—improved significantly by discharge. Results suggest that while some adolescents exhibit enduring anhedonia, pessimistic attributional style appears to be a concomitant feature of an acute depressive state.

## 1. Introduction

Anhedonia, the diminished capacity to experience pleasure, is of special interest in relation to major depression because diminished pleasure is a core manifestation of the disorder [1] and is associated with higher suicidal risk [2]. Anhedonia in adults is correlated with self-reported depressive measures and is associated with the diagnosis of depression but not other diagnoses [3]. Research with adults [3] has been consistent with Klein's hypothesis [4] about the existence of a qualitatively distinct subtype of major depression that has been variously defined as “truly anhedonic”, or “endogenomorphic.” It has been shown that adult patients with this subtype of depression are more severely depressed than depressed patients with a more normal pleasure capacity [5]. Recovered adult anhedonic depressed patients still had a lower pleasure score upon recovery than normally hedonic patients [5]. It is possible that some patients have a characterological anhedonic trait that predisposes them to endogenomorphic depressive states [5, 6]. In contrast to the patients with “endogenomorphic” depression, patients with situational depression have preserved hedonic capacity [7]. Yet recent studies showed that acute stress reduces reward responsiveness and had been linked to anhedonic behaviors thus raising the possibility of “situational” anhedonia [8]. 

Available research with children has demonstrated that anhedonia is consistently associated with the diagnosis of Major Depressive Disorder [9]. However, in contrast to adult studies, research with children has failed to find a correlation between self-report depression measures and anhedonia [9] or self-reported enjoyment and interest, possibly related to anhedonia [10]. Interpretation of these data remains unclear and can be related to the characteristics of the samples or developmental differences [9]. In particular, prepubertal children have lower capacity than adolescents or adults to report on their depressive symptoms [11].

Very little is known about anhedonia in depressed adolescents. In a study of nonreferred adolescents Carey et al. [12] did not find an association between self-reported depression and frequency of engaging in pleasant activities. On the other hand, it has been demonstrated that clinical picture of adolescent depression is more similar to adult depression, in particular, adolescents experience greater anhedonia compared to children [11, 13]. The concept concerning the existence of two qualitatively distinct subtypes of depression among adults (i.e. “exogenomorphic” or “situational” versus “endogenomorphic” or “truly anhedonic” depression corresponds to the notion of Garland and Weiss [14] who proposed that there may be two subgroups of adolescent depression: mild, situational, placebo-responsive depressions; and severe, genetically loaded, treatment-resistant depressions.

 Dysfunctional or pessimistic attributional style is closely related to such concepts as learned helplessness and external locus of control and, along with the anhedonia, has been accorded a pivotal role in conceptualizations of Depressive Disorder [15]. Attributional style is the manner in which a person explains the cause of negative and positive events. This concept has its roots in the learned helplessness model of depression and Beck's cognitive theory of depression [16]. Attributing the cause of uncontrollable bad events to internal, stable and global factors and to a lesser degree, the opposite style for attributing good events to external, unstable and specific factors, is a maladaptive or pessimistic attributional style. 

Pessimistic attributional style has been shown to be associated with depression in all age groups, and particularly in adolescents, for example, in a cross-sectional study by Gotlib et al. [17]. In our previous work [18] we have discussed the association between dysfunctional attributional style, sense of subjective incompetence and demoralization [19]. Our present study attempts to add to the literature by examining stability of pessimistic attributional style over the course of intensive inpatient treatment. 

 Recently published, by the American Academy of Child and Adolescent Psychiatry, Practice Parameter for the assessment and treatment of children and adolescents with depressive disorders [20] presented the prevailing view on the role of attributional style in childhood depression: “negative attributional style (is)… associated with poor outcome [21, 22]." "The onset and recurrences of Major Depression may be moderated by the presence of stressors. However, the effects of these stressors also depend on the child attributional styles." The very term “attributional style” implies that it is a permanent characteristic. 

However, the relationship between dysfunctional attributional style and Depressive Disorder appears to be complex and there is controversy among researchers. Abramson et al. [15] postulated that maladaptive attributional style is “depressogenic.” Lau and Eley [23] consider attributional style as a risk marker of genetic effects for adolescent depressive symptoms. Lewinsohn and colleagues [24] postulated that a bout of depression will lead to the development of a stable pessimistic explanatory style, a possibility that they have labeled the “scar hypothesis.” Nolen-Hoeksema [25] demonstrated in a longitudinal study with children in a nonclinical sample that after the first depressive episode attributional style became more dysfunctional than prior to the onset of depression, providing support for a scar hypothesis. There are several ways in which pessimistic style could develop during a depressive episode. The deficits in school performance and peer interactions that children often show when depressed could convince a child that he or she has low abilities, is unlikable, and generally is not able to control important outcomes in life [22]. 

Other research contradicts the scar hypothesis. In particular Asarnow and Bates [26] found in the sample of child psychiatric inpatients in the cross-sectional study that children with remitted depression have an explanatory style that was as optimistic as normal controls. In summary, while there is no doubt that pessimistic explanatory style is associated with depression, there remains a controversy in studies concerning the nature of the relationship between pessimistic style and depression, and whether pessimistic attributional style is a state marker of depression or a trait-like characteristic. We did not find studies addressing the “scar hypothesis” in adolescents with the treated depressive episode, that is, whether a depressive episode in treated adolescents leaves a “scar” such as a stable pessimistic explanatory style.

To date, there have been no longitudinal studies on adolescent anhedonia. The longitudinal studies on adolescent attributional style are very scarce [27, 28] and concerned with nonreferred, nontreated adolescents. These studies provided controversial results. In the study of Stevens and Prinstein [27], attributional style measured at Time 2 in 11 months since the outset of the study appeared to be a stable characteristic. Schwartz et al. [28] who measured attributional style in one year since the outset of the study found that “youth experienced significant changes in their attributional styles over time (from adaptive to maladaptive and vice versa).“ 

To our knowledge there have been no studies examining attributional style and anhedonia pre- and post-treatment in depressed adolescents. Further investigation of anhedonia and attributional style in adolescent depression can be of theoretical and practical importance. It is important to understand whether anhedonia relates to a subtype of depression among adolescents, and whether anhedonia and attributional style represent a clinical state or a trait. This understanding can be important for the prognosis at an acute phase, and for prognosis and treatment planning after the resolution of acute depressive symptoms.

The objective of the current study was to examine the relationship between self-reported depressive scores, clinical diagnosis of major depression, subsyndromal depression, anhedonia and attributional style in a sample of adolescent inpatients with depressive symptomatology at admission and at discharge.

Based on clinical impressions we hypothesized that many cases of depression in hospitalized adolescents are situational depressions and are phenomenologically similar to demoralization. We expected that pessimistic attributional style as well as anhedonia would be associated with the severity of self-reported depression and with the diagnosis of depression upon admission. The latter was based on a premise that demoralized patients may have an acute anhedonic state in the midst of a depressive episode. We hypothesized that both anhedonia and pessimistic attributional style would improve upon discharge.

## 2. Methods

### 2.1. Sample and Procedure

Eighty-one consecutive adolescent inpatients were screened for depressive symptomatology upon their hospital admission to the inpatient acute care adolescent psychiatric ward, during a five month period. Patients were administered the Beck Depression Inventory (BDI) [29] within three days of admission and their treating clinicians completed a Major Depressive Disorder (MDD) criteria sheet. The MDD criteria sheet is a checklist of the symptoms according to DSM-IV criteria for major depression, which includes a depression severity rating and duration criteria. MDD criteria sheet allows to document depressive symptoms elicited by clinician according to DSM-IV criteria for a Major Depressive Disorder. It had been used previously for the clinical and research purposes in the institution where study had been carried out (unpublished data). Patients who completed questionnaires and did not have mental retardation or neurological impairment were included. Four patients (5%) refused to participate, 4 mentally retarded patients were excluded and one patient was unable to participate because of an acute catatonic state at time of admission. None of the four patients who refused to participate was judged by their clinician as being depressed. The remaining 71 patients completed the BDI and their treating clinicians completed the MDD criteria sheet. Fifty-one of these 71 patients met the inclusion criteria of having a BDI score of 10 or above and/or having at least four symptoms on the MDD criteria sheet (three symptoms in addition to essential symptoms of depression or anhedonia) and were enrolled into the study. The decision to include patients with near threshold MDD was made based on the fact that subsyndromal depression causes significant functional impairment in adults [30] and adolescents [31]. It also provided an opportunity to compare patients who met full DSM-IV criteria for MDD with the patients who had only subsyndromal depression. Those patients who were enrolled were administered the Pleasure Scale for Children (PLS) [9] and the Children's Attributional Style Questionnaire—Revised (CASQ-R) [32] at admission (Time 1) and at the discharge (Time 2), and repeated BDI at the time of the discharge. The questionnaires were completed in one session in the presence of one of the investigators (Z.G. or R.E.). Some patients had very long hospital stay due to disposition problems. To increase comparability of the data patients whose discharge was delayed after remission of their symptoms due to placement problems were tested a second time one month after their admission. Clinicians were blind to the scores on the self-rated scales at the time of the completion of the MDD sheet. Final diagnosis was established based on DSM-IV criteria by the treatment team led by one of the investigators (R.D.), who were blind to the self-rated scales. Data were collected via chart review on subject's final psychiatric diagnosis, age, gender, ethnicity, and family composition. Major stressors were assessed based on parent, patient and clinician report. Thirty-nine patients (78%) had testing with the BDI, CASQ-R and PLS at the time of their discharge (Time 2). Patients who were tested at time 1 only (*N* = 12) had very short hospital stays and most of them were discharged precipitously against medical advice. Patients who have been tested at Time 1 and Time 2 had at least a one week interval between testing. Patients who had testing at Time 1 and at Time 2 did not differ on any sociodemographic variables from patients who had testing only at Time 1. Patients who were tested only at Time 1 tended to have less depressive symptoms and lower depression severity. 11 consecutive inpatients who did not have depressive symptomatology were tested upon admission only, with the BDI, CASQ-R and PLS and their clinicians completed MDD criteria sheet. These subjects constituted a comparison group and were included into some analyses. 

The study sample characteristics are described in the Table 1. 29 out of 36 patients (81%) with any acute Depressive Disorder (MDD being subsumed under Depressive Disorder group, Dysthymia being excluded) had comorbid diagnoses. Out of 27 patients with MDD 13 patients had comorbid diagnoses (48%). All fifteen patients who had concerning depressive symptomatology (BDI ≥ 10) but less than four symptoms on MDD criteria sheet had primary diagnoses other than Depressive Disorder. The study was approved by Long Island Jewish Medical Center Institutional Review Board. All subjects provided assent and parents or legal guardians provided consent.

### 2.2. Instruments

 We used Beck Depression Inventory to assess self-reported depressive symptomatology. BDI has been shown to be a reliable measure of self-reported depression in adolescents [33]. We chose the conventional cut-off score of 10 [33] to identify the presence of clinically concerning depressive symptoms. The primary measure for anhedonia was the Pleasure Scale for Children (PLS), the 39-item measure which was developed by Kazdin [9]. This questionnaire is a list of events and activities that children would normally find rewarding. The items reflect three categories similar to anhedonia scales for adults [3, 34]: physical anhedonia or activities involving physical pleasures (e.g. eating a favorite meal, lying in bed etc.); social anhedonia or activities involving other persons (e.g. talking to friend, being told how great one looks) and other activities that do not fall within the above (e.g. related to interest and achievement). The child or adolescent is asked to decide whether the activity would make him or her feel “very happy”, “happy” or “would not matter.” We made slight modifications of the scale to make language more appropriate to use with adolescents, for example, “special present" instead of “new toy" or “someone asks you to hang out with them" instead of "play". The primary measure for attributional style was Children's Attributional Style Questionnaire—Revised which is a shortened 24-item version [32] of the original 48-item questionnaire [35]. The CASQ, also referred as referred to as the KASTAN-CASQ [35], is the primary measure of attributional style for youth ages 8 to 18. Psychometric properties of the CASQ-R have been investigated by Thompson et al. [36] who concluded that it is a psychometrically adequate measure. The scale uses a forced choice format to assess causal attriibutions. Each item presents a hypothetical situation followed by two statements regarding why the event happened. Adolescents choose the response that best explains why they believe that event might have occurred. Three dimensions of attributions (i.e. internal-external, global-specific, stable-unstable) are assessed. An equal number of items assessed each dimension for both good and bad outcomes. Composite attributional style can be calculated based on all of the items for negative events (“composite negative") and all of the items for positive events (“composite positive"). A sample item that measures internality while holding stability and globality would be, for example,: “A good friend tells you that he hates you." Choices are (a) my friend was in a bad mood that day (external) (b) I was not nice to my friend that day (internal). Attributing negative events to internal, stable, and global factors and the opposite style for attributing positive events is associated with lower CASQ scores, reflective of less adaptive or more depressogenic attributions.

### 2.3. Data Analysis

Pearson correlations with one-tailed tests were computed for continuous variables. Independent samples *T*- tests were performed to assess group differences. Paired Sample *T*-tests were used to assess change over time on dependent variables. Partial correlation between Pleasure Capacity scores and CASQ scores while controlling for BDI was conducted. A separate analysis was performed for the patients with the most significant clinical improvement defined as a 50% decrease from the initial BDI score.

## 3. Results

Pleasure scale scores and CASQ-R scores showed significant inverse correlation with the BDI scores at Time 1 and Time 2, number of depressive symptoms and depression severity as rated by clinician (see Table 2). Independent sample *T*-tests demonstrated a significant difference in Pleasure scale scores between patients with the diagnosis of the Major Depressive Disorder (*N* = 27) ($\bar{x}\;=\;70.63\;\pm \;16.02$) and those without such diagnosis (*N* = 24) ($\bar{x}\;=\;80.71\pm 12.75$) (*t* = 2.46, *df* = 49, *P* < .01). Pessimistic attributional style was not associated with the diagnosis of Major Depressive Disorder.

CASQ-R scores were strongly associated with Pleasure scale scores (*r* = .69, *P* < .001) in the adolescents with a diagnosis of Depressive Disorder even when controlling for the BDI score; there was no association between those variables for patients without the diagnosis of Depressive Disorder (see Table 3).

 Differences in Pleasure Scale scores and CASQ-R scores at Time 1 and Time 2 were tested via Paired Sample *T*-tests, which indicated significant changes in CASQ-R scores and Pleasure Scale scores over time (see Table 4). Table 4 also summarizes attributions about bad events and good events at Time 1 and Time 2. 

A separate analysis performed for the patients with the most significant clinical improvement defined as a 50% decrease from the initial BDI score (*N* = 19) indicated even more significant increases in CASQ-R scores at the time of discharge approaching levels for normal epidemiological controls [35, Kaslaw, personal communication] ($\bar{x}=1.05\;\pm \;5.65$ at Time 1 versus $\bar{x}=\;4.26\pm 4.66$ at time 2; $\bar{x}=\;4.87\pm 3.39$ for normal epidemiological control. There was no difference in the change of the Pleasure Capacity over time among significantly improved patients and the rest of the study sample. 

Independent samples *T*-tests demonstrated significant differences on Pleasure scale scores and CASQ-R scores between the patients with depressive symptomatology (study sample, *N* = 51) and patients without depressive symptomatology (comparison group, *N* = 11) (see Table 5). Adolescents in the comparison group had higher mean scores on a Pleasure Scale and differed significantly from the depressed adolescents. Patients in the comparison group had significantly more optimistic attributional style than patients in the study sample. The difference in attributional style was across both dimensions, attributions for positive and for negative events. 

Scores on self-reported and clinician-rated measures did not correlate with sociodemographic variables except for a modest correlation between remote stressors and depression severity (*r* = .39, *P* < .01).

There was no significant difference on Pleasure Scale scores and CASQ-R scores between adolescents with “pure” MDD (*N* = 14) and those with MDD and comorbid disorders (*N* = 13).

## 4. Discussion

At admission, anhedonia and pessimistic attributional style were associated with self-reported depression, a number of depressive symptoms and depression severity. Anhedonia, but not pessimistic style, was associated with the diagnosis of Major Depressive Disorder. One possible explanation might be that while pessimistic style is associated with an overall level of psychological distress, it is not a core feature of MDD in adolescents. The finding that anhedonia is associated with the diagnosis of depressive disorder is consistent with previous research with adults [1] and children [9]. Our results contradict findings that anhedonia was not associated with self-reported depression in children [9] and with involvement in pleasurable activities by adolescents [12]. However, this finding is consistent with studies with adults that reported association between anhedonia and self-reported depression and depression severity [3]. Our results are intuitive and consistent with the notion that compared to depression in children, the clinical picture of adolescent depression is more similar to adult depression and is consistent with previous reports that adolescents experience greater anhedonia compared to children [11, 13]. We also believe that these results may have to do with the characteristics of our sample, that is, sample of seriously depressed hospitalized adolescents compared to non-hospitalized patients in the study of Carey et al. [12], and with the better ability of adolescents to report depressive symptoms. 

These findings have important clinical ramifications. Several studies for example,Fawcettet al. [2] have shown that severe anhedonia is associated with suicidal risk and may respond to pharmacotherapy and therapeutic interventions such as cognitive therapy. Klein [4] suggested that “truly anhedonic" depressions show better response to treatment with antidepressants than do other types of depressions. The results of our study suggest potential usefulness of assessing anhedonia as related to hospitalization and discharge criteria. 

There was a significant difference in anhedonia and attributional style between inpatients with and without depressive symptomatology. For attributional style, this difference was found across both dimensions, that is, attributions for positive and for negative events. Adolescents in the comparison group were much less anhedonic and more optimistic than patients in the study sample. These findings support further the association between both anhedonia and pessimistic style with depressive symptomatology. We do not have ready explanation for the fact that non-depressed inpatients were more optimistic than remitted depressed inpatients and had higher CASQ-R scores than normal epidemiological controls for children. It could be due to our small sample size or that remitted depressed adolescents may still have residual pessimistic traits. Also, one may speculate that non-depressed adolescent inpatients might have an abnormally optimistic style. More extensive research of non-depressed adolescent inpatients is needed and would clarify this issue. 

 It is intriguing that anhedonia and pessimism were strongly associated in adolescents with a diagnosis of any Depressive Disorder, even when controlling for BDI score. There was no association between those variables for patients without the diagnosis of Depressive Disorder. Nurcombe et al. [37] question if Depressive Disorder in adolescence is a distinct categorical entity—making an analogy to patients in a general hospital that have fever, but who no physician identifies as having "Major Fever Disorder." Our results are consistent with the findings of Kazdin [9] who demonstrated in a cross-sectional study that children high in anhedonia showed a dysfunctional attributional style. Those children showed less active involvement in seeking rewarding events, lower expectations for positive outcomes and more attributions for these outcomes. A strong association between anhedonia and helplessness in adolescents with the diagnosis of Depressive Disorder might suggest the possibility of a causal and bidirectional relationship between them and their involvement into pathogenic mechanism of depression. There is a striking similarity between the learned helplessness animal model and the more recently developed anhedonia model. In both models, animals are exposed to stressful stimuli, which are noxious stimuli in the learned helplessness model and chronic unpredictable stress in the anhedonia model [38]. This similarity raises questions that await future research, in particular if there may be a common pathway linking stressful stimuli to helplessness and anhedonia. 

Even though the study was not designed to formally validate the Pleasure Scale for use in adolescents, it provides some evidence of its validity. We have compared our data with the results of Kazdin [9]. The means are quite similar for non-depressed inpatient adolescents in our study and non-depressed inpatient children in the Kazdin sample (*x* = 84.8  versus 84.6, resp.) and anhedonia is slightly higher in depressed adolescents compared to depressed children (*x* = 75.4 versus 78.8, resp.), which is consistent with reports that depressed adolescents have greater anhedonia compared to children [11, 13]. 

Anhedonia improved over the course of patients' hospital stay but, contrary to our hypothesis, while statistically significant this change does not appear to be clinically meaningful (about a 3 point increase for the scale with the score range 39 to 117). There was no difference in change in anhedonia between significantly improved patients and the rest of the study sample. Our pre-study hypothesis was based on our expectation that most cases of adolescent depression are phenomenologically similar to demoralization; consistent with the notion by Garland et al. [14] that majority of the cases of adolescent depression are situational depressions. Patients with situational depression have preserved hedonic capacity, even though during acute state their demoralization can be superficially mistaken for an anhedonia [7]. The notion by Garland and Weiss [14] was based on the data on a nonreferred sample. It appears to be a feasible explanation that there is a much higher proportion of patients with non-situational depression in the sample of in-patient depressed adolescents than in nonreferred adolescents. Another possible explanation is that normally hedonic depressed patients actually do not show acute anhedonia in the midst of their acute depressive episode as measured by the pleasure scale. This would be consistent with Clark et al. [5] who found that normally hedonic adult depressed inpatients assessed on admission had Pleasure scores only slightly below those of normal subjects and below their own scores upon recovery.

Pessimistic style improved significantly (with about 1.7 point increase) and this improvement appears to be more clinically meaningful on a scale ranging from −12 to +12. This change affected both attributions for bad events and good events. Interestingly, improvement for attributions for positive events was more robust and approached the normal levels. One can speculate that while recovering from depression adolescents would still attribute bad outcomes to themselves but would be able to take credit for good outcomes. Patients who had a significant improvement in depression defined as a decrease in BDI of more than 50% from the initial score had even more impressive improvement in pessimism: with approximately a 2.7 increase in the score, approaching epidemiological normal controls. It is important to note that adolescents who showed significant improvement were as pessimistic upon admission as the rest of the sample. Scores upon discharge were approaching levels for the epidemiological controls, especially so for the significantly improved group. This is a finding that contradicts the scar hypothesis, which holds that depression leaves scars in explanatory style, and even recovered children never regain their optimism. Our findings are consistent with the study of Asarnow and Bates [26] who showed that remitted children inpatients were as optimistic as a never depressed group. Importantly, Asarnow and Bates [26] noted that the remitting depressive group was composed primarily of children whose depressions rapidly remitted following hospitalization and separation from their family and school environments. Limitation of that study was that data on initial (pre-treatment) scores were not available. Nolen-Hoeksema [25] argued that subjects in that study could have never had a pessimistic style. Our subjects had a demonstrated pessimistic style upon admission so this change did take place over the brief period of hospitalization. This is a fascinating finding which shows that explanatory style in adolescents is a flexible and dynamic feature. One might speculate that developing cognitions of adolescents with newly formed capacity for abstraction are very dynamic. This would make them more vulnerable to pessimism but also more capable of recovery. Depressed adolescents in our sample showed high capacity for remoralization in a protective therapeutic setting. The fact that pessimistic style did not discriminate between patients with the diagnosis of Major Depressive Disorder while anhedonia did probably means that anhedonia is a more primary, core characteristic of depression in adolescents and attributional style is less specific to the diagnosis of depression. It is conceivable that there is an overlap between constitutional factors reflected by anhedonia and demoralization reflected by pessimism. 

Lewinsohn et al. [39] in their prospective study of adolescent Major Depressive Disorder among nonreferred adolescents have shown that depressotypic attributional style predicted a future psychopathology in formerly depressed young men. Due to the nature of their sample, depressotypic cognitions have been measured in nontreated adolescents. Lewinsohn et al. [39] commented that important issues that need to be addressed in future research are whether depressotypic cognitions are measured at intake or at the end of treatment. Our findings further underscore the importance of this distinction. We suggest that future research should assess prognostic value of both pre-and post-treatment attributional style and perhaps the prognostic value of its change over the course of treatment. Further research is needed to assess change in pessimistic attributional style separately in hedonic and anhedonic depressed adolescents. 

Scores on self-reported and clinician-rated measures did not correlate with sociodemographic variables. This was contrary to our expectations, because one would think that depression might be associated with a number of major stressors or with an unfavorable family situation. That no relationship was found for family situation might be because the entire sample was disadvantaged in this regard, with only 30% of adolescents living in an intact biological family: there was not enough variability within the sample. It is also possible that the environmental component is not as decisive as factor in the development of adolescent depression. Similarly there was no association between recent stressors and depressive variables. However, there was a modest correlation between remote stressors and depressive severity (*r* = .39, *P* < .01). This is consistent with the findings of Nolen-Hoeksema [25] that negative life events play a larger role in the development of depression in earlier life, and later it is personal characteristics that play an important role, with life events becoming less important. 

Our data should be interpreted in the context of several limitations of our present study. The first limitation is the lack of normative data for adolescents on anhedonia. Second limitation is the small size of the study sample and a very small size of comparison group and heterogeneity of the study sample with the high number of comorbid disorders. Further research is needed to determine if these data can be replicated or generalized to other clinical populations for example, less severely disturbed adolescents. However comorbidity did not appear to affect Pleasure Scale scores and CASQ scores. Third limitation is that we did not use more stringent instruments such as Research Diagnostic Criteria to establish the diagnosis of Major Depressive Disorder. Another limitation is that we did not have further follow-up data on our subjects and did not investigate if improvement in attributional style was lasting or related to a protective milieu and was thus short-lived.

## 5. Conclusions

We suggest that anhedonia is a critical characteristic of adolescent depression, one that could be intimately involved in the pathogenic mechanism of the depressive episode through association with pessimistic attributional style; also there may be a subgroup of depressed adolescents who exhibit enduring anhedonia, which might create vulnerability to future bouts of depression. 

Our results suggest that contrary to the “scar hypothesis,” pessimistic attributional style is not an enduring trait in depressed adolescents but rather a concomitant feature of an acute depressive state. Our findings suggest that hospitalized depressed adolescents may have superimposed demoralization that is highly responsive to intensive inpatient psychiatric treatment with its protective supportive therapeutic milieu.

Our findings underscore the importance of maintaining treatment gains such as improvement in optimism. Further research should have longitudinal designs, in particular including post-episode data.

## Figures and Tables

**Table 1:** Description of the sample (*N* = 51).

Mean age, y (SD)	14.9 (1.59)
Male, *n* (%)	14 (27.5)
Ethnicity, *n* (%)	
Caucasian	35 (69)
African-American	11 (22)
Hispanic	3 (6)
Asian	1 (2)
Indian	1 (2)
Family status, *n* (%)	
Living with both biological parents	16 (31)
Living with a single biological parent	20 (39)
Living in stepfamilies	8 (16)
Living in a foster family	6 (12)
Living in a group home	1 (2)
Mean length of stay, days (SD)	18.22 (25.45)
Primary Diagnosis, *n* (comorbid with depressive Disorder)	
MDD	27
Other depressive disorder: depressive disorder	9
NOS or adjustment disorder with depressed mood	
Bipolar disorder	11 (0)
ADHD	6 (2)
ODD	10 (8)
Anxiety disorder	10 (7)
Eating disorder	3 (2)
Dysthymia	4 (0)
Substance abuse	7 (4)
Psychosis	11 (6)

**Table 2:** Correlation coefficient between measures.

Scores on independent measures, Time 1	BDI, Time 1	BDI, Time 2	Number of depressive symptoms	Depression severity
Pleasure Scale	−.40**	−.33*	−.38**	−.32*
CASQ-R, Total	−.59***	−.48***	−.32*	−.27*
CASQ-R	.55**	.45**	.22	.21
CO-NEG				
CASQ-R				
CO-POS	−.52**	−.42**	−.35**	−.27*
Number of stressors in last 6 months	−.08	.02	.02	.20
Number of remote stressors	−.03	.16	.20	.39**

*P < .05; **P < .01; ***P < .001.

**Table 3:** Correlation between attributional style and pleasure capacity.

Patients with depressive disorder		Patients without depressive disorder		
(*N* = 36 for Time 1; *N* = 28 for Time 2)		(*N* = 15 for Time 1; *N* = 11 for Time 2)		
	PLS, Time 1	PLS, Time 2	PLS, Time 1	PLS, Time 2
CASQ, Time 1	.69***	.51**	.28	−.11
CASQ, Time 2	.50**	.47**	.47	.00

**P < .01; ***P < .001.

**Table 4:** Difference over time.

	Normative data (scale range)	$\bar{x}$ (SD) Time 1	$\bar{x}$ (SD) Time 2	*t*	*P*
BDI	< 10 (0–63)	20.92 ± 13.16	11.15 ± 9.56	5.56	<.001
PLS	No normative data (39–117)	75.37 ± 5.30	78.16 ± 14.29	−1.76	<.05
CASQ-R	4.87 ± 3.39 (−12 to +12)	1.69 ± 5.29	3.36 ± 5.01	−2.83	<.01
CASQ-R CO-NEG	≈2.7 ± 1.9 (0 to −12)	4.55±2.78	3.79 ± 2.76	2.04	<.05
CASQ-R CO-POS	≈7.6 ±2.2 (0 to + 12)	6.24±3.11	7.15 ± 3.06	−2.52	<.01

**Table 5:** Group comparison.

	Non-depressed controls (scale range)	*x* (SD) Study sample (*N* = 51)	*X* (SD) Non-depressed inpatients ( *N* = 11)	*t*	*P*
BDI	≤ 10 (0–63)	20.92 ± 13.16	3.27 ± 3.23	−8.47	< .001
PLS	No normative data (39–117)	75.37 ± 15.30	84.82 ± 15.85	1.85	< .05
CASQ-R	4.87 ± 3.39 (−12 to +12)	1.69 ± 5.29	6.36 ± 2.69	−2.83	< .001
CASQ-R CO-NEG	≈2.7 ± 1.9 (0–12)	4.55 ± 2.78	2.6 ± 1.8	−2.18	< .05
CASQ-R CO-POS	≈7.6 ± 2.2 (0–12)	6.24 ± 3.11	9.0 ± 1.7	2.84	< .01
